# Lutembacher Syndrome with Sinus Venosus-Type Interatrial Communication: An Educational Presentation

**DOI:** 10.21470/1678-9741-2021-0323

**Published:** 2022

**Authors:** Andréa Bezerra de Melo da Silveira Lordsleem, Sandro Gonçalves de Lima, Lucas Soares Bezerra, Eveline Barros Calado, Fabio Antônio Amando Granja, Brivaldo Markman-Filho

**Affiliations:** 1 Cardiology Service, Hospital das Clínicas/Universidade Federal de Pernambuco (HC/UFPE), Recife, Pernambuco, Brazil.; 2 Postgraduate Program in Therapeutic Innovation, Centro de Biociências, Universidade Federal de Pernambuco, Recife, Pernambuco, Brazil.

**Table:** Abbreviations, Acronyms & Symbols

ASD	= Atrial septal defect
IAC	= Interatrial communication
LA	= Left atrium
LS	= Lutembacher syndrome
LV	= Left ventricle
RA	= Right atrium
RV	= Right ventricle

## INTRODUCTION

Lutembacher syndrome (LS) was first described by Johann Friedrich Meckel in 1750. In 1811, Corvisart described the association of atrial septal defect (ASD) with mitral stenosis. However, the first comprehensive report of these two defects was made by Rene Lutembacher in 1916, and the syndrome was named after him.

LS is a condition in which there is a combination of mitral valve stenosis and ASD^[1]^. LS with a sinus venosus-type interatrial communication (IAC) in association with anomalous pulmonary vein drainage is very rare.

A 42-year-old female patient presented to a cardiology outpatient clinic due to dyspnea on light exertion, associated with palpitations, during which she had nausea and chest discomfort. She had suffered with plegia and muscle atrophy in her right upper limb since she was three years old, which was possibly attributed to poliomyelitis sequel. She reported no cough, orthopnoea, or paroxysmal nocturnal dyspnea.

**Fig. 1:**
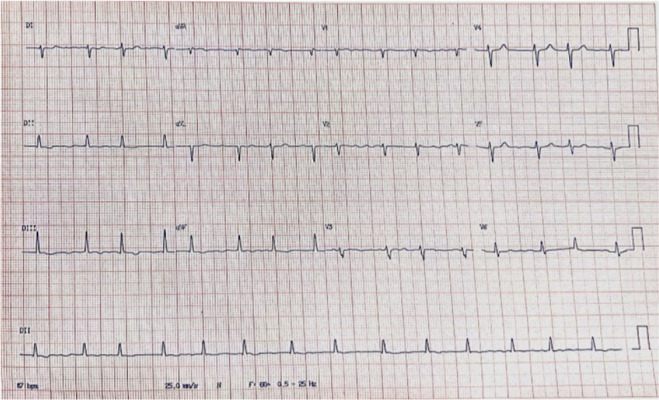
Eletrocardiogram demonstrating atrial fibrillation with an axis deviation to the right.

A physical examination revealed an irregular heart rhythm, jugular turgor, a diastolic murmur in the mitral area (2+/6+), and a systolic murmur in the tricuspid area (3+/6+). The electrocardiogram showed atrial fibrillation with an axis deviation to the right (Figure 1).

**Fig. 2:**
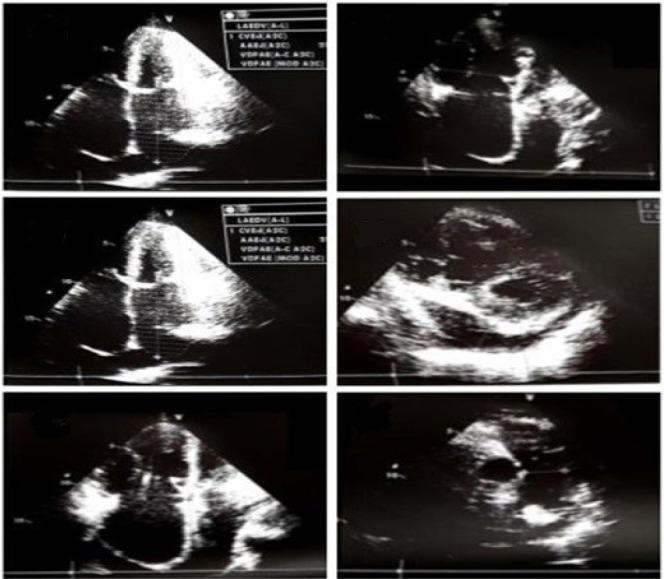
Transthoracic echocardiogram demonstrating an opening in the dome of the mitral valve with significant mitral stenosis, biatrial and right ventricular enlargements, moderate/significant tricuspid insufficiency, mild pulmonary arterial hypertension and pericardial effusion.

The patient also had a transthoracic echocardiogram showing an opening in the dome of the mitral valve with significant mitral stenosis, biatrial and right ventricular enlargements, moderate/significant tricuspid insufficiency, mild pulmonary arterial hypertension, and pericardial effusion with no hemodynamic repercussions (Figure 2).

**Fig. 3:**
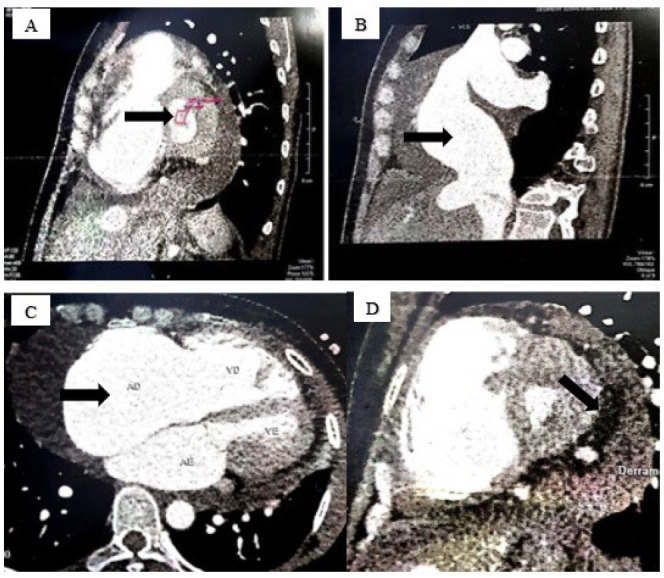
Angiotomography demonstrating: A - mitral valve with thickened leaflets and reduced opening (valve area 0.6 cm2); B - a significantly enlarged biatrial and of the right ventricle; C - a significantly enlarged of the inferior vena cava and D - a large pericardial effusion.

A coronary angiotomography was requested to investigate the possibility of associated coronary artery disease, which demonstrated pericardial effusion, a discrete myocardial bridge in the middle third of the anterior descending artery with no luminal reduction, thickened mitral valve leaflets and reduced opening (estimated valve area at 0.6 cm2), and the presence of ground-glass images in both lung fields (Figure 3).

During surgical exploration, the mitral valve presented with very thick leaflets and a commissural fusion promoting severe stenosis. The subvalvular apparatus was fused with intense fibrosis, with no technical conditions for the preservation of the native valve.

**Fig. 4:**
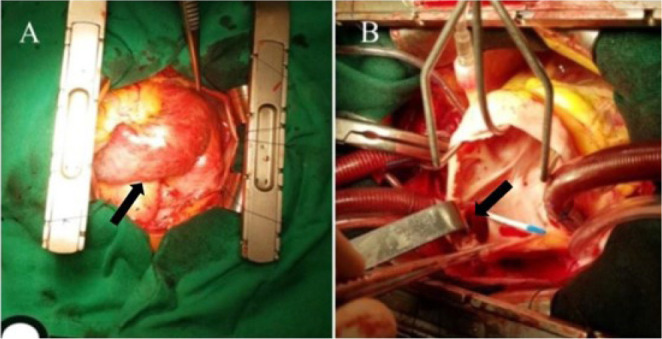
In A we observe a significant enlargement of the right chambers and the superior vena cava. In B we may observe the sinus venosus-type IAC.

In the surgical procedure for valve replacement with a 27-mm mechanical prosthesis and tricuspid commissuroplasty using the Kay technique to correct tricuspid insufficiency, superior sinus venosus-type IAC was identified with superior pulmonary vein return to the superior vena cava. The IAC was closed, and the pulmonary vein was diverted to the left atrium (Figure 4).

## QUESTIONS

- What is the cause of mitral valve stenosis and ASD?
- What would be considered as a clinical challenge?
- What are the criteria for the indication of surgical treatment?
- What is the challenge for the treatment of mitral valve disease and what is the justification for valve replacement with a metallic prosthesis?
- Considering the neurological features, any other etiology should be suspected?

### Discussion of Questions

Histopathological examination of the excised mitral valve and fragment of the biopsied pericardium identified severe valve fibrosis, mesothelial hyperplasia in the pericardium, and non-specific inflammatory signs. A course of colchicine was given during the postoperative period to treat the pericardial effusion.

Question A. LS is described as an association of mitral valve stenosis and ASD^[1]^. Amongst the ASDs, the most prevalent is the ostium secundum-type IAC^[2]^. Both defects, ASD and mitral stenosis, may be congenital, acquired, or iatrogenic, secondary to transseptal puncture during mitral valvuloplasty^[3]^. In addition, they may also present hemodynamic repercussions that vary according to the development time of the disease, size of the ASD, and severity of the mitral valve disease^[3]^. Sinus venosus-type IAC is present in only five to 10% of cases, and its relationship with LS has rarely been described in the literature^[2^,^4^,^5]^. In the case reported, the etiology of mitral stenosis was secondary to chronic rheumatic disease, given the high prevalence of this disease in our country and the echocardiographic characteristics of rheumatic involvement, despite the absence of specific histopathological changes of rheumatic involvement. The defect of the interatrial septum was considered to be congenital due to the absence in the patient’s history of data that could suggest an acquired etiology.

Question B. The main peculiarity was that the patient presented an association between mitral stenosis, sinus venosus-type IAC, and anomalous pulmonary vein return. In classic LS cases, left atrial and pulmonary vein hypertension do not occur, since the left atrium is able to decompress through the septal defect^[6]^. With this pathophysiology, in LS there is an attenuation of the clinical manifestations of mitral stenosis and an increase in IAC, *i.e.*, the symptoms and signs of pulmonary congestion disappear and the pulmonary hyperflow increases, with a predisposition for developing pulmonary arterial hypertension^[8]^. As pulmonary hypertension progresses, the right ventricle becomes hypertrophic and, therefore, less compliant, which decreases or eliminates left-to-right shunting. Consequently, there is an increase in pressure in both atria, significantly increasing the diastolic gradient across the mitral valve^[8]^.

Question C. The patient presented with symptomatic and significant mitral stenosis associated with a large pericardial effusion and an important tricuspid valve insufficiency. Thus, with clear surgical indication.

Question D. In women still in the reproductive phase, biological prosthesis was considered, but the heart time option was for metallic prosthesis due to permanent atrial fibrillation.

**Fig. 5:**
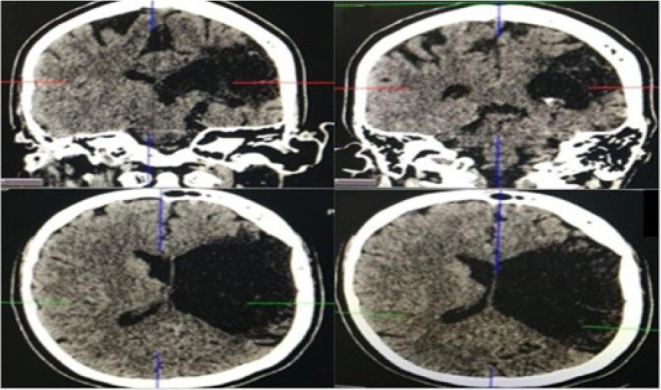
Cranial computed tomography scan with lesions suggestive of chronic ischemia in the left parietal, temporal and frontal lobes.

Question E. Considering that the neurological sequel presented by the patient could be of embolic vascular origin secondary to heart disease and not due to poliomyelitis, as believed at admission, because it was only in the right arm, a cranial computed tomography scan was performed. This examination revealed lesions suggestive of chronic ischemia in the left parietal, temporal, and frontal lobes, thereby confirming the suspicion of vascular etiology (Figure 5).

## BRIEF CONSIDERATION OF THE CASE REPORTED

LS is a condition in which there is an association of mitral valve stenosis with ASD. LS with sinus venosus-type IAC associated with anomalous pulmonary vein return is very rare. During cardiac surgery for mitral valve replacement with a mechanical prosthesis and tricuspid commissuroplasty, upper sinus venosus-type IAC was identified with a right superior pulmonary vein return into the superior vena cava.

A surgical approach is the traditional therapy for the treatment of LS^[9]^. In this case, the patient was referred for surgery given her condition of significant pericardial effusion and severe mitral stenosis, presenting with good clinical development after the procedure.

## LEARNING POINTS

- Although very rare, the presence of sinus venosus-type IAC is a variation of LS.
- LS represents a challenge for clinical diagnosis due to the masking of the signs and symptoms of mitral stenosis by shunt through the defect of the interatrial septum.
- Surgical therapy is highly important for the treatment of LS and can be associated with histopathological analysis.
- Neurological symptoms should be suspected as a vascular sequel of the disease.

**Table:** Authors’ Roles & Responsibilities

ABMSL	Substantial contributions to the conception of the work; and the acquisition, analysis, and interpretation of data for the work; drafting the work and revising it critically for important intellectual content; final approval of the version to be published
SGL	Substantial contributions to the conception of the work; and the acquisition, analysis, and interpretation of data for the work; drafting the work and revising it critically for important intellectual content; final approval of the version to be published
LSB	Substantial contributions to the conception of the work; and the acquisition, analysis, and interpretation of data for the work; drafting the work and revising it critically for important intellectual content; final approval of the version to be published
EBC	Substantial contributions to the conception of the work; final approval of the version to be published
FAAG	Substantial contributions to the conception of the work; final approval of the version to be published
BMF	Substantial contributions to the conception of the work; final approval of the version to be published
